# Assessment of the quality and variability of health information on chronic pain websites using the DISCERN instrument

**DOI:** 10.1186/1741-7015-8-59

**Published:** 2010-10-12

**Authors:** Jatin Kaicker, Victoria Borg Debono, Wilfred Dang, Norman Buckley, Lehana Thabane

**Affiliations:** 1 Department of Anesthesia, Michael G. DeGroote School of Medicine, McMaster University, 2U1-1200 Main Street West, Hamilton, Ontario, L8N 3Z5, Canada; 2 Department of Clinical Epidemiology and Biostatistics, McMaster University, 1200 Main Street West, Hamilton, Ontario, L8N 3Z5, Canada; 3 Biostatistics Unit, Father Sean O'Sullivan Research Centre, 3rd Floor Martha, Room H325, St. Joseph's Healthcare Hamilton, 50 Charlton Avenue East, Hamilton, Ontario, L8N 4A6 Canada

## Abstract

**Background:**

The Internet is used increasingly by providers as a tool for disseminating pain-related health information and by patients as a resource about health conditions and treatment options. However, health information on the Internet remains unregulated and varies in quality, accuracy and readability. The objective of this study was to determine the quality of pain websites, and explain variability in quality and readability between pain websites.

**Methods:**

Five key terms (pain, chronic pain, back pain, arthritis, and fibromyalgia) were entered into the Google, Yahoo and MSN search engines. Websites were assessed using the DISCERN instrument as a quality index. Grade level readability ratings were assessed using the Flesch-Kincaid Readability Algorithm. Univariate (using alpha = 0.20) and multivariable regression (using alpha = 0.05) analyses were used to explain the variability in DISCERN scores and grade level readability using potential for commercial gain, health related seals of approval, language(s) and multimedia features as independent variables.

**Results:**

A total of 300 websites were assessed, 21 excluded in accordance with the exclusion criteria and 110 duplicate websites, leaving 161 unique sites. About 6.8% (11/161 websites) of the websites offered patients' commercial products for their pain condition, 36.0% (58/161 websites) had a health related seal of approval, 75.8% (122/161 websites) presented information in English only and 40.4% (65/161 websites) offered an interactive multimedia experience. In assessing the quality of the unique websites, of a maximum score of 80, the overall average DISCERN Score was 55.9 (13.6) and readability (grade level) of 10.9 (3.9). The multivariable regressions demonstrated that website seals of approval (*P *= 0.015) and potential for commercial gain (*P *= 0.189) were contributing factors to higher DISCERN scores, while seals of approval (*P *= 0.168) and interactive multimedia (*P *= 0.244) contributed to lower grade level readability, as indicated by estimates of the beta coefficients.

**Conclusion:**

The overall quality of pain websites is moderate, with some shortcomings. Websites that scored high using the DISCERN questionnaire contained health related seals of approval and provided commercial solutions for pain related conditions while those with low readability levels offered interactive multimedia options and have been endorsed by health seals.

## Background

### Health information and the Internet

The Internet is an important international electronic network and mass medium for individuals seeking information pertaining to almost any topic, including health information and health care services [1]. Reports by the National Telecommunications and Information Administration in the United States indicate that in 2000, 44% of individuals had access to the Internet [2]. This percentage increased to 54% by 2008, with current usage estimated at over one billion users in the world [3-5]. In 2009, Statistics Canada found that 80% of Canadians over the age of 16 use the Internet for personal reasons with searching for health information reported by 70% of home user, up from 59% in 2007 [6]. Growth in internet usage has led to about 37% of consumers using the electronic network to retrieve health related knowledge [7]. Consumers report convenience, diversity and anonymity of information sources on the Internet as reasons for using it as attractive alternatives to consulting with a clinician [8]. One concern and public health issue is the quality of health information on the Internet. Despite efforts toward standardization, health information on the web remains unregulated and varies substantially in quality, accuracy, and readability [9].

### Issues with online health information

An individual's risk of encountering an online website that is compromised in its quality, accuracy and readability is a combination of two variables: the proportion of inadequate information on the web, and the competency of the population to filter out those sites that lack reliability and validity. It is plausible that a correlation exists between the proportion of websites presenting low quality health information and adverse health outcomes in a target population. Consumer risk can be reduced through the introduction of clear critical appraisal tools and with a standardized website evaluation system for health information, thus improving the ability of users to locate trustworthy sites and to filter the inadequate ones [1].

Apart from the quality of health information on the web, patients also find many websites presenting health information using highly technical language. Technical presentations may be advantageous for researchers and clinicians; however, this technical language can be overwhelming and confusing, especially if it is not properly explained [3]. Therefore, it is also imperative to systematically assess the presentation of online health information using readability algorithms to ensure that such information is easily assessable to lay audiences.

### The Internet and pain management

Chronic pain is a serious health and socioeconomic concern [10]. In the mid 1990s, the National Population Health Survey in Canada showed that about four million Canadians, about 17% of the population at the time, suffered from chronic pain with a negative impact on quality of life [11]. Seventy percent of those reporting pain rated their pain as moderate to severe, intruding upon activities of daily living. Patients with chronic pain visited their family physician more frequently (12.9 versus 3.8 mean visits per year) and spent more time as hospital inpatients (3.9 versus 0.7 days) than those without pain [12]. In the United States, the American Productivity Audit found that over a two-week period in 2001, 13% of the workforce experienced loss in productivity due to chronic pain [12]. This report highlighted that 76% of the production time loss was due to diminished performance of staff suffering from pain, rather than absenteeism [12].

Many chronic pain patients look to the Internet to learn more about their condition, treatment and prognosis. However, a great deal of online information about pain may be inaccurate in one or more elements. An investigation at a pain clinic in the Netherlands found that patients felt confident about the credibility of the information ascertained from online resources, and as a result only half of the patients discussed the knowledge acquired from websites with physicians [13]. This practice of unconditionally accepting online health information can be harmful as another study, from the University of Western Australia, found that chronic pain information on the Internet is poor in quality [14]. The majority of the 27 websites that were evaluated were rated as fair or poor. This study mentions that though there may be high quality chronic pain content available online that is relevant and reliable, it is difficult to find amongst all the content that is of poor quality [14]. To assist patients in the process of selecting quality sites, health related websites should be judged by both the quality of health information presented and design features that facilitate or impede use. The DISCERN quality index tool is one way to evaluate the reliability and quality of online health information and treatment choices [15].

### The DISCERN questionnaire

Several solutions have been proposed to address the issues of accuracy, quality and reliability of information found on websites. These solutions have included electronic filtering of web-based information, creation of ethical codes of conduct for providers of web-based information (currently done on a voluntary basis) and assessment of websites by health professionals [16,17]. An additional approach is to design and publicize instruments to assess the reliability and utility of information found on websites. This allows consumers to appraise web-based information themselves [17]. The DISCERN questionnaire is a valid and reliable instrument for analyzing written consumer health information. It is the first standardized quality index of consumer health information that can be used as a critical appraisal tool to evaluate health information by not only health professionals, but also by patients and the general population. This questionnaire was derived systematically with the input of an expert panel, health information providers and patients from a self-help group [15]. Thus, the purpose of this study was to determine the quality and readability of pain management websites and factors that can explain variability in the quality and grade level readability scores.

## Methods

### Search strategy

A list of search terms commonly used by patients suffering from pain was obtained from published literature. These terms included "Pain", "Chronic Pain", "Back pain", "Arthritis", and "Fibromyalgia" [18]. Each of these keywords were entered into three different search engines (Google, Yahoo, and MSN) chosen because of their popularity [19]. The first 20 links reported by each search engine per keyword were evaluated for quality using the DISCERN questionnaire and readability through the Flesch-Kincaid Algorithm [9,20].

### Inclusion/exclusion criteria

Websites were included in the investigation if they provided detailed information pertaining to the five search terms and treatment options for patients suffering from the conditions. Websites that were unrelated to pain or only provided a list of website links were removed. Sponsored links and banner advertisements were excluded as they are normally ignored [9]. Websites that were considered 'For profit' were included only if they attempted to educate patients about pain conditions, and presented products with scientific evidence. 'For profit' websites were excluded: if their only intention was to sell a product, if the site promised quick and unrealistic dramatic results, made claims that one remedy will cure a variety of illnesses through some miraculous breakthrough, or used excessive sensational writing [21].

### DISCERN questionnaire scoring criteria

The DISCERN questionnaire consists of 16 questions, on a continuous rating scale of 1 to 5, where 1 = definite NO and 5 = definite YES. Any rating in between (such as a 2, 3 or 4) suggests that some of the elements asked of by the question are present to a certain extent. These questions are categorized into three sections [22]. Section 1 (questions 1 to 8) assesses reliability, dependability and trustworthiness of a website; Section 2 (questions 9 to 15) focuses on the quality of information about treatment choices; and Section 3 (question 16), evaluates overall quality rating on a continuous rating scale for the online website with a ratings of 1 = Low to 5 = High. Rating for question 16 is done independently of the rating given for the other previous 15 questions.

### Readability

The readability of an online webpage refers to the level of reading difficulty found in written passages [20]. Two factors comprise the readability of an online passage: 1) average sentence length in words -- the average of the numeric word count found in a passage and 2) average word length in syllables -- the number of syllables per 100 words. A lower readability score found using the Flesch-Kincaid algorithm indicates an easier reading level. Readability was assessed using the cross-platform, open source Java Flesch 2.0 Software [23].

### Data abstraction

The first stage of this investigation involved determining attributes found on websites. A random sample of 25 websites was assessed by the three reviewers to determine the characteristics most commonly present on webpages. It was decided that each subsequent website would be evaluated on potential for commercial gain, website seals of approval (for example, Health on Net), language(s) and multimedia (for example, online videos, audio recordings). The number of websites presenting these characteristics or lacking them was recorded. Each website was also evaluated on quality, as indicated by the DISCERN score tabulated and grade level readability, based on the Flesch-Kincaid algorithm. Overall and characteristic specific average DISCERN and readability scores were also calculated, with means (standard deviation (SD)), presented.

This investigation had three reviewers assess the quality of online health information. VBD and JK assessed the online information from Google, VBD and WD assessed Yahoo, and JK and WD assessed the information found on MSN. Each reviewer independently assessed the same list of websites and generated a DISCERN Score which was averaged and used for statistical purposes. In order to assess the level of agreement in the DISCERN rating scores between the reviewers; the chance corrected agreement (weighted kappa) value was generated [24].

### Statistical analysis

The effect of each website characteristic on the variability in DISCERN and readability scores were assessed using univariate and multivariable analyses. No *a priori *hypotheses were generated for the effect of each website characteristic on quality and readability scores. Thus, the analysis in this study is primarily exploratory and meant to generate hypothesis for further investigation in larger studies. Model assumptions were assessed using the Normal Probability PP and QQ plot and by examining the residuals.

Website characteristics were compared independently to DISCERN and readability scores using univariate regression analyses. The characteristics were selected for inclusion in the multivariable analysis using alpha = 0.20 during the univariarate analysis. Characteristics from the univariate analyses that were of interest were further investigated by a multivariable regression using alpha = 0.05. The results were reported as estimates of the model coefficient Beta, corresponding to the 95% Confidence Interval (CI) and associated *P-*values. All *P-*values are reported to three decimals places with less than 0.001 reported as *P *< 0.001. Finally, for this investigation, reviewer agreement on website data abstraction was quantified using the weighted kappa statistic from the MedCalc (Version 11.3) Statistical Software [Mariakerke, Belgium] [25]. Descriptive and regression analyses were carried out using SPSS software Version 17 (Chicago, IL, USA).

## Results

### Average DISCERN and readability scores

**Figure 1:**
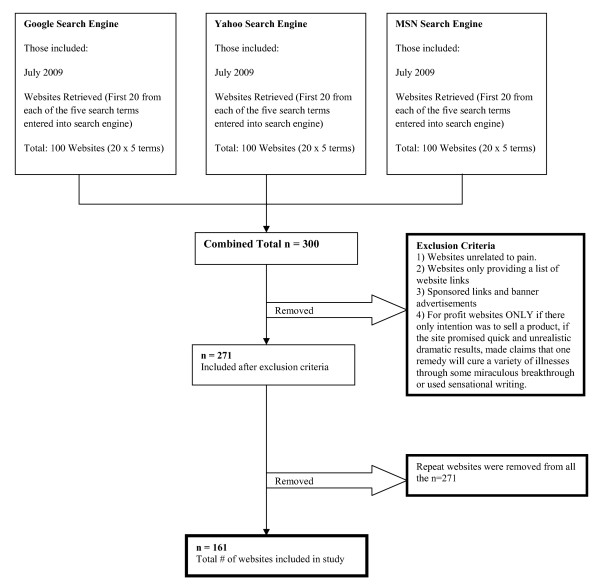
**Consort diagram, websites included in study**.

A total of 300 websites were reviewed in July 2009, 100 for each of the three search engines (Google, Yahoo and MSN). A total of 271 out of 300 websites were eligible for examination after application of the exclusion criteria (Figure 1). Removal of 110 duplicate websites left a total of 161 unique sites. The Google search engine contributed 55.3% (89/161 websites) of the unique sites evaluated with 22.6% from each of the Yahoo and MSN engines (36/161 websites). When examining the number of websites classified under each of the characteristics evaluated it was found that 6.8% (11/161 websites) of the websites offered patients commercial products for their pain related condition (Potential for Commercial Gain) and 36.0% (58/161 websites) presented a health related seal of approval. Similarly, 75.8% (122/161) of the websites were presented information in English only and 40.4% (65/161 websites) offered an interactive multimedia experience to viewers (Table 1). The mean (SD) DISCERN value for Google was 58.2 (12.4), Yahoo 60.0 (11.5) and MSN 53.2 (14.1). When examining average grade level readability using the Flesch-Kincaid algorithm, scores were found to be 11.1 (4.1) for Google, 10.5 (4.0) for Yahoo and 11.2 (3.6) for MSN. Grade level readability is associated with grades 1 to 8 correlated to students in elementary school, grades 9 to 12 for those in secondary (high) school and grades above 12 being collegiate.

**Table 1:** Descriptive table: n = 161 (total number of unique websites included)

Variable	Statistic N (% of total number of unique websites)
**Search engine**	
Google	89 (55.3)
Yahoo	36 (22.4)
MSN	36 (22.4)

Potential for commercial gain	11 (6.8)

Health related seals of approval	58 (36.0)

**Language**	
English only	122 (75.8)
English and another language	39 (24.2)

Multimedia	65 (40.4)

In assessing the quality of the unique websites, of a maximum score of 80, the overall mean DISCERN Score was 55.9 (13.6) and readability (grade level) of 10.9 (3.9). The mean DISCERN/Readability score for each of the four characteristics evaluated was also calculated (Tables 1 and 2). Finally, the inter-rater reliability for Google and Yahoo was moderate (қ = 0.58 and қ = 0.46) respectively. For MSN, the inter-rater reliability was қ = 0.64, a strength of agreement considered good.

**Table 2:** Summary of statistics of the DISCERN scores and readability

Website characteristic	Descriptive characteristics	
**Variable**	**DISCERN score **(Maximum Score of 80)	Readability score (grade level)*
	
	**Mean (SD)**	**Mean (SD)**

***Potential for commercial gain***		

Yes, n = 11	62.0 (8.0)	11.97 (2.5)

No, n = 150	55.4 (13.8)	10.85 (4.0)

***Health related website seals of approval***		

Yes, n = 58	59.5 (10.9)	10.3 (3.2)

No, n = 103	53.8 (14.5)	11.3 (4.2)

***Language***		

Only English, n = 122	56.1 (13.4)	10.8 (3.6)

English and another language(s), n = 39	54.9 (14.4)	11.2 (4.7)

***Multi-media***		

Yes, n = 65	55.8 (13.9)	10.40 (3.2)

No, n = 96	55.9 (13.4)	11.28 (4.2)

SD = standard deviation

*Based on the educational system of Ontario

Elementary School: grades 1 to 8

Secondary (High) School: grades 9 to 12

Collegiate (Post Secondary Education): above grade 12

### Factors associated with variability in DISCERN and readability scores

The univariate regression analysis for DISCERN scores of websites found both potential for commercial gain (*P *= 0.121) and health related seals of approval (*P *= 0.010) met the alpha = 0.20 criteria for the multivariable analysis (Tables 1 and 3). Using the multivariable analysis for DISCERN scores, a significant result was found for websites with health related seals of approval (*P *= 0.015) in comparison to websites that had a potential for commercial gain (*P *= 0.189) (See Tables 1 and 3).

**Table 3:** Univariate and multivariable results using total quality DISCERN scores as an outcome

Website characteristic	Univariate		Multivariable	
**Variable**	**Coefficient (95% CI)**	***P***	**Coefficient (95% CI)**	***P***

Potential for commercial gain	6.6 (-1.8, 15.0)	0.121	5.5 (-2.7,13.8)	0.189
		
Health related seals of approval	5.7 (1.4, 10.0)	0.010	5.4 (1.1, 9.8)	0.015

Language (English or English and another language)	-1.2 (-6.2, 3.7)	0.631		
		
Multimedia	-0.2 (-4.5, 4.2)	0.936		

CI = confidence interval

Similarly, for grade related readability values of websites, the univariate analysis revealed that both health related seals of approval (*P *= 0.109) and websites that had multimedia options (*P *= 0.155) were eligible for multivariable analysis. The multivariable analysis for readability determined that online material containing seals of approval (*P *= 0.168) and websites that possessed a form of interactive multimedia (*P *= 0.244) contributed to lower readability scores, although both were not statistically significant (See Tables 1 and 4).

**Table 4:** Univariate and multivariable results using readability grade level scores as an outcome

Website characteristic	Univariate		Multivariable	
**Variable**	**Coefficient (95% CI)**	***P***	**Coefficient (95% CI)**	***P***

Health related seals of approval	-1.0 (-2.3, -0.2)	0.109	-0.9 (-2.2, 0.4)	0.168
		
Multimedia	-0.9 (-2.1, 0.3)	0.155	-0.7 (-2.0, 0.5)	0.244

Potential for commercial gain	1.1 (-1.3, 3.5)	0.356		
		
Language (English or English and another language)	0.4 (-1.0, 1.8)	0.596		

CI = confidence interval

## Discussion

The Internet has the potential to rapidly provide both patients and health care providers with access to health information. With its growing use there is increasing concern about the quality of online health information, as well as variability amongst health websites [13,16,24]. The American Medical Association (AMA) has published guidelines to aid patients in search of health information on the Internet and they also address the variability in patient health literacy, quality of content and access to information online [16,26]. It is therefore imperative to use a consistent approach to the assessment of online health information commonly found by patients. In this investigation, the DISCERN questionnaire was used to evaluate the quality of websites and the Flesch-Kincaid algorithm was used to assess grade level readability.

The mean DISCERN score for the 161 unique websites was 55.9 of a maximum of 80 with a SD of 3.9. This suggests information of moderate quality, with potentially important but not serious shortcomings. The majority of websites found were above the sixth grade reading level recommended for patient directed literature as a mean Readability Grade Level Score was found to be 10.9 (3.9), an intermediate grade level (see Tables 1 and 2) [27].

The potential implications of this investigation for clinical practice, patient care and protocols pertaining to online health information are widespread. Although only the relationship between website seals of approval and DISCERN scores was found to be significant, several factors contribute to higher DISCERN and lower readability scores. Beta coefficient values suggest that potential for commercial gain and health related seals of approval result in high DISCERN scores while lower readability is associated with health related seals of approval and interactive multimedia.

Health related seals of approval are attempting to unify the quality of medical information on the Internet and offers the audience an indication of the online provider's commitment to providing quality information [28]. The coefficient beta values found in this investigation for seal of approval in regards to both the DISCERN and the readability scores suggests that it is advantageous for more health websites to strive for a seal of approval certification and for patient/health care providers to seek such sites. Lower readability scores were correlated with websites offering interactive multimedia. This may occur because when developing information for multiple media, information must be reviewed on numerous occasions with the resulting information presented more succinctly. Finally, those websites engaged in selling commercial products have a need to present a clear sales message and therefore may achieve high DISCERN scores. This enables patients (potential consumers) to understand their medical conditions better and make informed decisions for their treatment options. The observations of this study would suggest that patients attempting to obtain health information online should start by selecting websites that are interactive, display seals of approval, are easy to navigate and provide commercial treatment options. We realize that this latter recommendation may be controversial because of the conflict of interest inherent in the use of health related information to promote product sales. On the other hand, it is to the advantage of the commercial entity to have its products used correctly by the appropriate patient population.

In terms of uniqueness, this is the first study that analyzes pain websites using the highly reliable DISCERN tool and also provides novel insight into the variability of quality and readability scores when examining online websites. Previous studies using the DISCERN tool have focused on the quality of websites for pain [29], low back pain [30,31], rheumatoid arthritis [32,33], burn scar management [34], and treatment for cough in children [35] have used different tools. Similar to this investigation, these studies repeatedly reported that websites on the Internet are of moderate quality, and are not consistent in keeping pace with new research.

Despite attempts towards the standardization of online health information, only 32.2% of websites reviewed presented with a health related seals of approval. This investigation found health related seals of approval to not only be an important factor in high DISCERN scores, assessing website content and treatment options but also the most significant factor for low grade level readability scores. In addition to health related seals of approval, interactive multimedia options, offered in 40.4% of websites were found to be a significant factor in low readability scores. Finally, while only 6.8% of the websites evaluated offered a commercial solution for patients with pain conditions, that factor contributed most to high DISCERN scores.

Websites are constantly being updated or removed and new ones are emerging, all of which may change the quality of websites found in this study. For practical reasons, only websites in English were considered. It may be advantageous to investigate search engines in other languages to determine if any discrepancies exist in the quality of the online health information. Only the top 20 websites were investigated for each chronic pain search term. It may be worthwhile to compare the quality of online health information from the top 50 websites and not just the top 20 as done here from each search engine, as quality may vary. Finally, the DISCERN instrument is an effective tool for assessing the quality of online information as it pertains to treatment, but less effective in evaluating other website information.

## Conclusion

The overall quality of chronic pain websites is moderate, with some shortcomings that need to be addressed. We have found that websites which contain health related seals of approval and offer information for alterative commercial solutions to pain related conditions have higher DISCERN scores. Lower readability levels were again found in websites with health related seals of approval and also interactive multimedia options.

## Abbreviations

AMA: American Medical Association.

## Competing interests

The authors declare that they have no competing interests.

## Authors' contributions

JK was involved with the conception of the study, participated in the study design, the acquisition of data, performed the statistical analysis, and drafted the manuscript. VBD helped conceive the study, participated in the design of the study, assisted with data acquisition and helped revise the manuscript. WD was involved with data acquisition and revisions to the paper. NB made substantial contributions to the concept and design of the study, and critically revised the manuscript. LT helped conceive the study and made substantial contributions to the statistical analysis, interpretation of data and critically revised the manuscript. All authors read and approved the final manuscript.

## Pre-publication history

The pre-publication history for this paper can be accessed here:

http://www.biomedcentral.com/1741-7015/8/59/prepub
